# Advancing radiology practice and research: harnessing the potential of large language models amidst imperfections

**DOI:** 10.1093/bjro/tzae022

**Published:** 2024-08-14

**Authors:** Eyal Klang, Lee Alper, Vera Sorin, Yiftach Barash, Girish N Nadkarni, Eyal Zimlichman

**Affiliations:** Division of Data-Driven and Digital Medicine (D3M), Icahn School of Medicine at Mount Sinai, New York, NY, 10029-6504, United States; The Charles Bronfman Institute of Personalized Medicine, Icahn School of Medicine at Mount Sinai, New York, NY, 10029-6504, United States; Tel Aviv University School of Medicine, Tel Aviv University, Tel Aviv, 69978, Israel; Department of Radiology, Mayo Clinic, Rochester, MN 55905, United States; Tel Aviv University School of Medicine, Tel Aviv University, Tel Aviv, 69978, Israel; Department of Diagnostic Imaging, Sheba Medical Center, Ramat Gan, 52621, Iarael; The Sheba Talpiot Medical Leadership Program, Sheba Medical Center, Ramat Gan, 52621, Israel; Division of Data-Driven and Digital Medicine (D3M), Icahn School of Medicine at Mount Sinai, New York, NY, 10029-6504, United States; The Charles Bronfman Institute of Personalized Medicine, Icahn School of Medicine at Mount Sinai, New York, NY, 10029-6504, United States; Tel Aviv University School of Medicine, Tel Aviv University, Tel Aviv, 69978, Israel; The Sheba Talpiot Medical Leadership Program, Sheba Medical Center, Ramat Gan, 52621, Israel; Hospital Management, Sheba Medical Center, Ramat Gan, 52621, Israel

**Keywords:** large language models, natural language processing, ChatGPT, artificial intelligence

## Abstract

Large language models (LLMs) are transforming the field of natural language processing (NLP). These models offer opportunities for radiologists to make a meaningful impact in their field. NLP is a part of artificial intelligence (AI) that uses computer algorithms to study and understand text data. Recent advances in NLP include the Attention mechanism and the Transformer architecture. Transformer-based LLMs, such as GPT-4 and Gemini, are trained on massive amounts of data and generate human-like text. They are ideal for analysing large text data in academic research and clinical practice in radiology. Despite their promise, LLMs have limitations, including their dependency on the diversity and quality of their training data and the potential for false outputs. Albeit these limitations, the use of LLMs in radiology holds promise and is gaining momentum. By embracing the potential of LLMs, radiologists can gain valuable insights and improve the efficiency of their work. This can ultimately lead to improved patient care.

## Introduction

Large language models (LLMs) have recently advanced the field of natural language processing (NLP). NLP is a subset of artificial intelligence (AI) that enables computers to analyse human language. The introduction of ChatGPT, a chatbot powered by an LLM, has gained much interest. This is due to its ability to simulate human-like conversations. LLMs have a wide range of potential uses in radiology. Notably, they may transform the way we analyse text data by enabling a level of language comprehension that was previously unattainable.

## Understanding NLP

NLP is a domain in AI aimed at bridging a gap between human free-language communication and computer “understanding.”^1^ NLP has become increasingly important in recent years, with the huge surge of online information and the growing need for automated analysis of large datasets.^1^ One challenge with NLP is the complexity of comprehending human language. Words can mean different things depending on how they are used. They can also be written or said differently in different languages.

**Figure 1.**
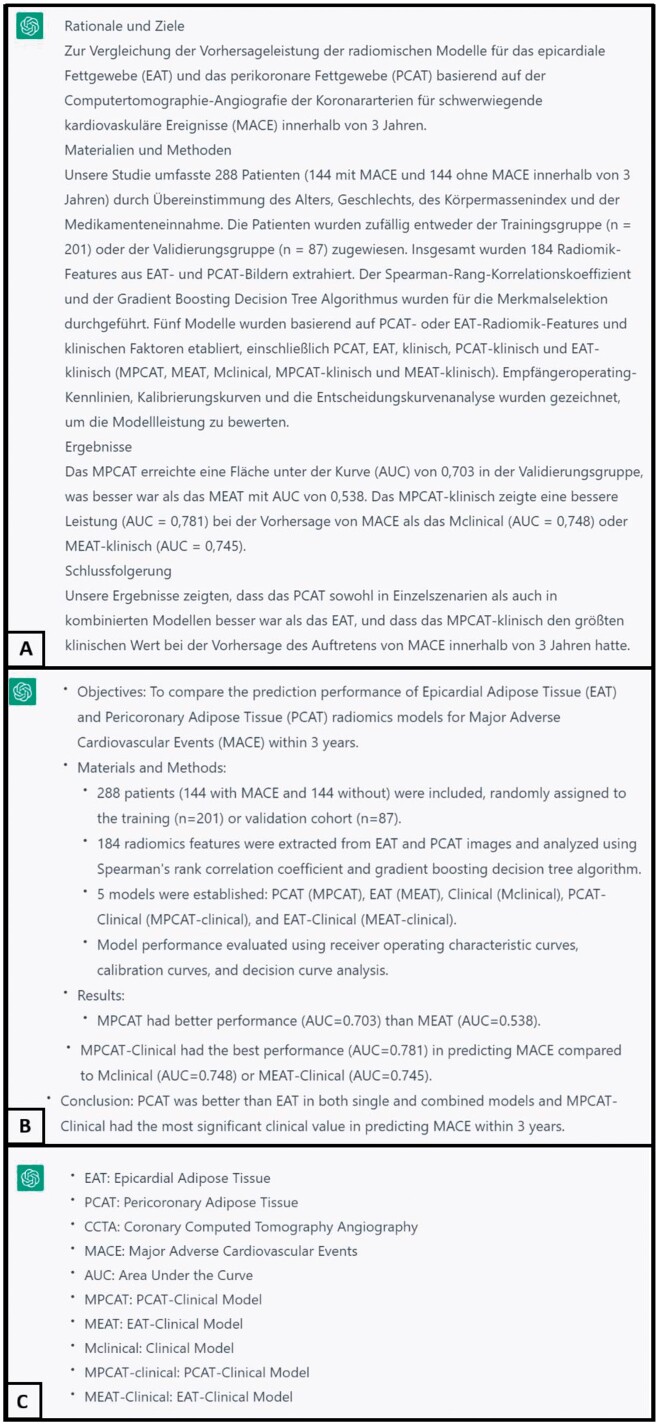
Assignments performed by ChatGPT. (A) Translation of text from English to German. Although ChatGPT performs well in translating academic texts, it may exhibit inaccuracies and difficulty translating certain words. (B) A summary of text into bullet points. ChatGPT can effectively identify the main aspects of the text and focus on critical information. (C) Identification and explication of all acronyms in the text.

NLP encompasses a variety of algorithms that can interpret and understand free-text data. Traditional NLP methods rely on statistical and rule-based techniques, which are labour-intensive to construct and apply, and they exhibit limited capabilities. In contrast, deep learning models primarily use artificial neural networks. These frameworks have the advantage of self-optimization and adaptability.^1^ Some examples of NLP applications include language translation,^2^ text summarization,^3^ voice recognition systems,^4^ and chatbots development that can engage in dialogues with humans (Figure 1A–C). In the context of radiology for instance, automatic text analysis may enable extraction of meaningful data from radiology reports.^5^^,^^6^

## Recent advances in NLP

In recent years, several types of deep learning algorithms for NLP have been developed. These algorithms generally adhere to a set of fundamental principles. The algorithms are usually composed of multiple connected units, which are akin to logistic regression units, that together compose a multi-layered artificial neural network.^7^

Initially, a deep learning algorithm may be provided with data accompanied by predefined answers. During the training phase, it independently devised methods to accurately interpret the data. Subsequently, the algorithm undergoes testing with unfamiliar data, applying patterns recognized during training to analyse this new information.^5^

### Attention mechanisms and transformers

A recent breakthrough in NLP is the introduction of the Attention mechanisms and the Transformer architecture. These novel algorithms were first introduced by Vaswani et al in 2017 in the paper “Attention is All You Need.”^8^ Attention mechanism allows the neural network to focus on specific parts of the input and ignore irrelevant information.^5^ The Transformer architecture stacks multiple layers of attention mechanism to process an input. Unlike traditional models that process data sequentially, Transformers handle data in parallel. Transformers have achieved state-of-the-art performance across a wide range of NLP tasks. A notable example is language translation, where accurately capturing the context and meaning of words and phrases is crucial.^8^

### Large language models (LLMs)

Following the advent of the Transformer and Attention architectures, numerous transformer-based models have been developed. More recently, LLMs have marked a significant breakthrough. LLMs are advanced algorithms constructed with multiple layers of transformer architectures, each layer containing millions of parameters, cumulatively reaching billions. These models excel in processing and generating text through a series of encoder and decoder mechanisms that capture and utilize contextual relationships within data. Specifically, LLMs leverage attention mechanisms to weigh the importance of different words in a sentence, allowing for more precise generation and understanding of language. They are designed to handle datasets scaling to terabytes, efficiently generating coherent and contextually appropriate text.

Training methods for LLMs involve diverse datasets sourced from publicly available texts, licensed corpora, and domain-specific databases. Data curation for these models includes preprocessing steps such as tokenization, normalization of text, and removal of sensitive information to comply with privacy standards.

Machine learning techniques including supervised, unsupervised, semi-supervised learning paradigms, and reinforcement learning from human feedback (RLHF) are employed to process the training data, depending on the model’s intended application. Supervised learning requires labelled datasets where input data are tagged with correct answers, whereas unsupervised learning finds patterns in unlabelled data. Semi-supervised learning combines both techniques, utilizing a smaller set of labelled data alongside a larger pool of unlabelled data. RLHF involves training models based on user interactions and feedback to refine responses and improve accuracy. This multifaceted approach enables training LLMs to adapt to various text-based tasks.

Notable LLMs include OpenAI’s GPT (Generative Pre-training Transformer), Meta’s LLaMA, Google’s Gemini, and Anthropic’s Claude. These LLMs have enabled advancements in complex NLP tasks. They generate human-like text for applications including question-answering, text summarization, content creation, and language translation. For example, LLMs can interpret and summarize medical documents or research papers. Furthermore, they demonstrate capabilities in logical and mathematical reasoning and show initial multimodal abilities that include image analysis, potentially useful for integrating textual and imaging data in radiology. This breadth of applications underscores the transformative impact of LLMs in NLP, with a huge potential to enhance medical research and patient care.

## Potential for enhancing clinical workflow and research in radiology

The introduction of LLMs may significantly enhance both academic and clinical aspects of radiology (Table 1). LLMs can analyse and interpret large amounts of text data in a short amount of time.^5^

**Table 1.** Benefits and limitations of current large language models.

Benefits	Limitations
Ability to quickly analyse and interpret vast amount of radiology reports	Generated text may sometimes be fake, termed “hallucinations,” requiring careful human consideration
Facilitates extraction of relevant information from articles and compare them to radiology reports texts	Reliant on quality and quantity of training data, may not accurately analyse or interpret data outside of its training
Develop NLP-based tools (eg, chatbots, voice assistants) to improve natural language communication in radiology	Can perpetuate disparities in healthcare
Analyse large amounts of data from clinical trials, leading to a better understanding of the safety and efficacy of new radiology technologies	Model security may not be adequate and could be subjected to adversarial attacks
Identify the evolution of radiology research over time, providing insight into the field's development and trends	Cannot be used as an academic writing tool. Further training on medical texts (eg, PubMed) is required
Improve patient comprehension of radiology reports	Privacy concerns arise as LLMs often process extensive personal or confidential information, necessitating robust data protection mechanisms
Flag important findings from radiology reports	Automation bias, the over-reliance on LLM decisions, can lead to errors, especially when users trust automated insights without sufficient scrutiny
Prioritize imaging requests and generate referrals based on clinical notes	

Abbreviations: LLMs = large language models; NLP = natural language processing.

In the context of research, the models can assist in the extraction of relevant information from academic articles and the identification of trends and patterns in radiology reports. These can be applied to enhance radiology research. For example, an LLM could be trained on a large dataset of radiology reports and identify common patterns that may not be immediately apparent to human readers. This could potentially lead to a more efficient and accurate analysis of radiology data, ultimately improving patient care.^5^

LLMs could also be used to identify trends in academic papers. This potentially could uncover insights that may be missed by traditional methods of analysis. Another potential benefit is the ability of LLMs to quickly index large amounts of text data, potentially improving the quality of research. For example, a recently published study by Khraisha et al^9^ evaluated GPT-4's performance when screening and extracting data from manuscripts in multiple languages. Guo et al^10^ used Chat GPT and GPT-4 for screening titles and abstracts based on predetermined inclusion and exclusion criteria to be included in a literature review. They compared the LLMs’ performance against human-reviewed papers across 6 review papers, screening over 24 000 titles and abstracts. The LLM accuracy reached 0.91 compared to human benchmark.^10^

As for potential clinical applications in radiology, LLMs can summarize reports, flag important findings, prioritize imaging requests, and generate referrals for examinations^11^; translate reports into various languages; and simplify reports for patient comprehension. For example, Le Guellec et al demonstrated that Version 1.3 of the Vicuna 13-B model, which is an open source model, have achieved a sensitivity of 96% and specificity of 98.9% for categorizing head MRI as normal or abnormal on a subset of 2398 radiology reports.^12^ In another study, Berigan et al compared various methods to deploy LLMs for creating patient-friendly radiology reports.^13^ Barash et al demonstrated encouraging results when using LLMs to generate radiology referrals and determine imaging protocols based on clinical cases.^11^

LLMs capabilities could assist radiology professionals by answering queries, providing information, or facilitating tasks, potentially reducing their workload, decreasing human errors, and allowing them to concentrate on more complex diagnostic challenges.

## Limitations of LLMs

While LLMs have the potential to impact academic research and clinical workflow in radiology, it is important to note their limitations (Table 1). LLMs rely on the quality and quantity of the training data used. They may not be able to accurately interpret data that falls outside the scope of their training. Another concern is the potential for LLMs to generate misleading or incorrect information, termed “hallucinations.”^14^ Given the high stakes of medical research and patient care, any text produced by LLMs must be subjected to thorough review by domain experts to ensure its accuracy and reliability. The inherent inability to trace these models’ decision-making process is a challenge for responsible integration in healthcare. Additionally, the use of LLMs in research writing raises concerns about plagiarism, as their output might unintentionally replicate existing texts without proper attribution, compromising the integrity of scholarly work. Careful human consideration is needed in any text creation by an LLM. The models can perpetuate disparities in healthcare. Especially in the healthcare domain, privacy concerns arise as LLMs often process extensive personal or confidential information, necessitating robust data protection mechanisms. Another limitation of LLMs is automation bias, the over-reliance on LLM decisions, can lead to errors, especially when users trust automated insights without sufficient scrutiny. Finally, LLM models security may not be currently adequate and could be subjected to adversarial attacks.^15^

## Call for action for radiology researchers

As a journal dedicated to advancing the field of academic radiology, the *British Journal of Radiology* has consistently been a leader in the search of innovative approaches to research and clinical practice. By leveraging the power of LLMs, radiologists can gain valuable insights and improve the quality of their clinical and academic work. Ultimately, this can lead to improved patient care.

However, the integration of LLMs in radiology faces several challenges. Concerns range from the inherent limitations of LLMs as discussed above, to their effective integration into existing radiology information systems, while maintaining patient data privacy and adhering to regulatory compliance. There is also a learning curve associated with understanding these models and effectively using the technology in a clinical context.

To address these challenges, radiologists must be informed about recent advancements. This may involve individual learning, institutional support, and collaboration with AI experts. Radiologists should be actively involved in the development of guidelines for the ethical use of LLMs. They should supervise and be part of these models’ integration and ensure their responsible implementation. Regulatory agencies should cooperate with healthcare professionals when establishing standards and protocols for the safe and effective clinical use of these technologies.

Finally, before any implementation is initiated, research is imperative for the evaluation of various potential use cases. Studies on these models’ performance in various clinical scenarios will provide valuable insights into the practical challenges and benefits of LLMs in practice.

Embracing the potential of LLMs requires a proactive approach, addressing their limitations and taking part in the ongoing evolution of technology. By actively participating in this endeavour, the radiology community can lead the upcoming revolution in healthcare.

In conclusion, LLMs have the potential to significantly enhance both academic research and clinical care in radiology through their ability to analyse and interpret large amounts of text data. LLMs have limitations, such as the reliance on the quality and quantity of training data, and the risk of generating false outputs. Yet, their impact on radiology could be tremendous and warrants further investigation.
